# Letter to the Editor

**DOI:** 10.1111/anec.12755

**Published:** 2020-03-03

**Authors:** Gerardus J. Hassing, Michiel J.B. Kemme, Pim Gal

**Affiliations:** ^1^ Centre for Human Drug Research Leiden The Netherlands; ^2^ Department of Cardiology Amsterdam UMC Amsterdam Cardiovascular Sciences Vrije Universiteit Amsterdam Amsterdam The Netherlands

With great interest, we read the article recently published in Annals of Noninvasive Electrocardiology by Majeed et al. (2019) The authors reported a significant positive association between total bilirubin (TB) and the uncorrected QT interval in an elderly, multiracial cohort. These findings also relate to early‐phase drug development, where healthy volunteers receive novel drugs and where the effect of these compounds on the QT interval is of interest for the compound developers. However, several key aspects should be taken into account when interpreting the results of the study by Majeed et al. (2019).

Firstly, physiologically the QT interval is correlated to the heart rate. Subsequently, appropriate interpretation of QT interval data requires a correction for heart rate. Fridericia's formula is used most often and was previously demonstrated to be adequate in liver cirrhosis patients. (Fridericia, 2003; Zambruni et al., 2007) The corrected QT and RR intervals were not reported throughout the article, which means that the association between the uncorrected QT interval and TB levels may have been caused solely by an association between the heart rate and TB levels.

Secondly, the interpretation of the association between the TB levels and the QT interval is affected by the population in which the study is executed. Majeed et al. used a pool of healthy volunteers and liver cirrhosis patients, (Majeed et al., 2019) making it a confounder in the analysis, thereby difficult to control for the potential other effects that liver cirrhosis and the severity of the liver cirrhosis may have on the QT interval.

With these remarks in consideration, we applied the analysis to our own dataset. Data from 1588 healthy male and female volunteers aged 18–30 years (mean age 22.7 ± 3.0) collected at our center, specialized at early‐phase drug development studies, were analyzed. Subjects considered healthy after a full medical screening were included in the analysis as described elsewhere. (Hassing et al., 2019) Mean TB was 0.69 ± 0.38 mg/dl with a range between 0.11 and 2.45 mg/dl. The QTcF interval was 406 ± 18 ms in the lowest quartile (TB < 0.47 mg/dl) compared to 402 ± 17 ms in the highest quartile (TB >0.82 mg/dl). In the univariate analysis, the uncorrected QT interval (standardized coefficient (SC) = 0.004, *p* = .879) was not associated with TB levels. In contrast, ventricular rate (SC = −0.058, *p* = .040) and the QTcF interval (SC = −0.071, *p* = .005) were significantly associated with TB levels. Note that the association between the QTcF interval and TB levels is negative as opposed to the positive association as reported by Majeed et al. The associations persisted in the multivariate analysis after controlling for calcium and potassium levels, body mass index and systolic blood pressure.

In conclusion, the association between the ventricular repolarization and TB levels depends on both the variables for which the QT interval is controlled and the subject population in which the analysis is performed.

## CONFLICT OF INTEREST

The authors declare that there are no conflicts of interest regarding this study.
